# Supplementary material for: Spatio-Temporal Variation and Influencing Factors of the Coupling Coordination Degree of Production-Living-Ecological Space in China
Source: Int J Environ Res Public Health. 2022 Aug 20;19(16):10370. doi: 10.3390/ijerph191610370 (PMC9407956; doi:10.3390/ijerph191610370)
Supplement: Supplementary file 1 [file ijerph-19-10370-s001.zip › ijerph-1842594-supplementary.pdf]

Spatio-Temporal Variation and Influencing Factors of the Coupling Coordination Degree of Production-Living-Ecological Space in China

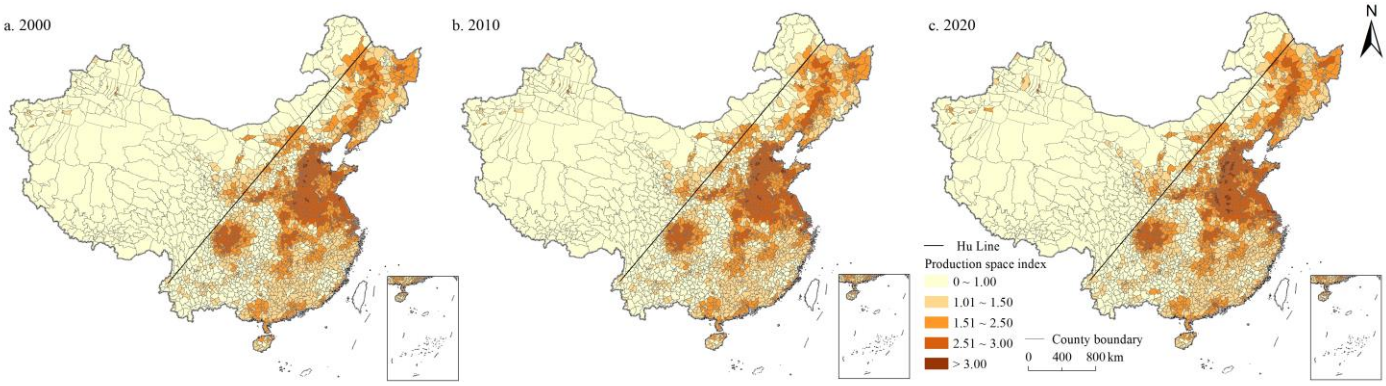

Figure S1. Spatial distribution of PSI in China during 2000—2020

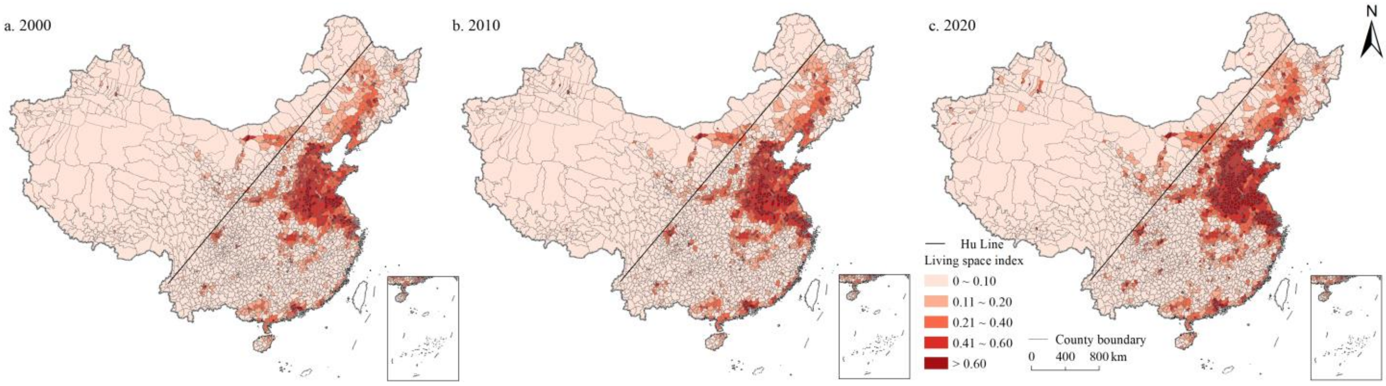

Figure S2. Spatial distribution of LSI in China during 2000—2020

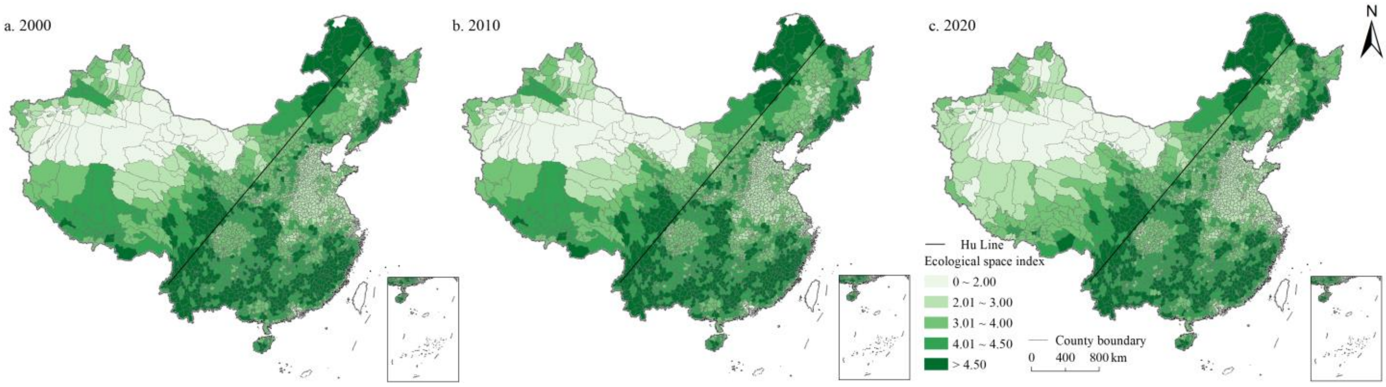

Figure S3. Spatial distribution of ESI in China during 2000—2020
